# An RND transporter in the monoterpene metabolism of *Castellaniella defragrans*

**DOI:** 10.1007/s10532-018-9857-6

**Published:** 2018-10-17

**Authors:** Edinson Puentes-Cala, Jens Harder

**Affiliations:** Dept. of Microbiology, Max Planck-Institute for Marine Microbiology, Celsiusstr. 1, 28359 Bremen, Germany

**Keywords:** RND efflux pump, Monoterpene, Toxicity, Anaerobic metabolism, *Castellaniella defragrans*

## Abstract

The betaproteobacterium *Castellaniella defragrans* 65Phen grows on monoterpenes at concentrations toxic to many bacteria. Tolerance mechanisms include modifications of the membrane fatty acid composition and the mineralization of monoterpenes. In this study, we characterized an efflux transporter associated to the monoterpene metabolism. The inner-membrane transporter AmeD (apolar monoterpene efflux) affiliated to the HAE3 (hydrophobe/amphiphile efflux) family within the Resistance-Nodulation-Division (RND) superfamily. RND pumps of the HAE3 family are known for transporting substrates into the periplasm. AmeD is co-expressed with the outer membrane protein AmeA and the periplasmic proteins AmeB and AmeC, suggesting an export channel into the environment similar to HAE1-type RND exporters. Proteins AmeABCD are encoded within a genetic island involved in the metabolism of acyclic and cyclic monoterpenes. The deletion of *ameABCD* translated into a decrease in tolerance to monoterpenes in liquid cultures. The addition of acetate as cosubstrate in limonene-containing cultures partially alleviated monoterpene toxicity in the deletion mutant. Accumulation of Nile Red in cells of *C. defragrans* required dissipation of the proton motive force with carbonyl cyanide *m*-chlorophenylhydrazone (CCCP). Cells lacking AmeABCD accumulated more Nile Red, suggesting an export function of the proteins. Our observations suggest that the tetrapartite RND transporter AmeABCD acts as an exporter during monoterpene detoxification in *C. defragrans*.

**Electronic supplementary material:**

The online version of this article (10.1007/s10532-018-9857-6) contains supplementary material, which is available to authorized users.

## Introduction

Monoterpenes are a diverse group of volatile biogenic hydrocarbons found mainly in the essential oils of plants. Produced as secondary metabolites, these compounds exhibit a myriad of biological functions such as pollinator attraction, plant–plant communication and as antimicrobials (Mahmoud and Croteau 2002). Due to their hydrophobic nature, monoterpenes tend to accumulate in cellular membranes altering the proton gradient, the electron transport and the stability of membrane proteins (Abrahim et al. 2003; Brennan et al. 2012; Griffin et al. 1999). Microorganisms using monoterpenes as carbon and energy sources have evolved mechanisms to circumvent this toxicity. Changes in membrane fluidity, monoterpene biotransformation and active secretion count among such adaptations (Bicas et al. 2008; Ramos et al. 2002; Ultee et al. 2000). Several efflux pumps of the Resistance-Nodulation-Division (RND) superfamily are reported to confer tolerance towards monoterpenes and other hydrocarbons (Kieboom et al. 1998; Segura et al. 2012). The RND efflux transporters MexAB-OprM and MexCD-OprJ have shown to be essential for growth of *Pseudomonas aeruginosa* exposed to monoterpene constituents of the tea-tree oil (Papadopoulos et al. 2008). Similarly, the complex AcrAB-TolC and several mutants thereof increased tolerance and enhanced monoterpene production in engineered *E. coli* strains (Dunlop et al. 2011; Foo and Leong 2013). Typically, RND efflux transporters active on volatile hydrocarbons belong to the hydrophobe/amphiphile efflux-1 (HAE1) family (Eswaran et al. 2004; Garcia et al. 2010; Nikaido 2011; Tseng et al. 1999). Members of this family are mostly tripartite consisting of an inner membrane substrate/proton antiporter, an outer membrane pore and a periplasmic membrane fusion protein (MFP). The latter links the inner and outer membrane components and facilitates substrate transport across the periplasm straight into the extracellular environment. Substrate specificity is determined by the inner membrane RND pump which recruits substrates from the periplasm or from the outer leaflet of the inner membrane (Daury et al. 2016; Nikaido 2011).

**Fig. 1:**
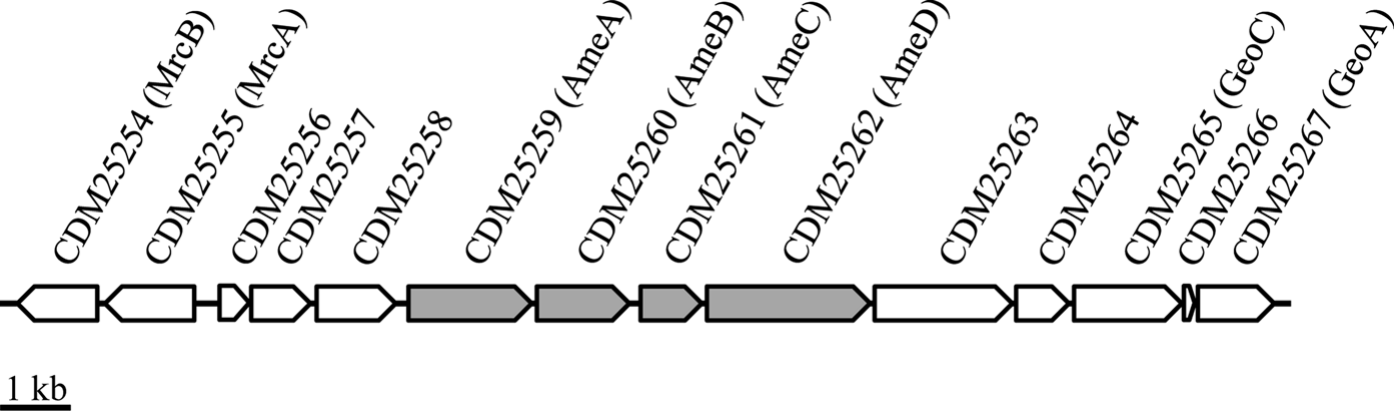
Gene cluster of the putative RND transporter AmeABCD (grey) and its genetic neighbors within the genomic island specialized in monoterpene metabolism: upstream the monoterpene ring cleavage operon *mrcABCDEFGH* and downstream parts of the cyclic monoterpene metabolism (*geoA* and *geoC*)

The betaproteobacterium *Castellaniella* (ex *Alcaligenes*) *defragrans* 65Phen mineralizes several monoterpenes under denitrifying conditions (Foss et al. 1998) and tolerates concentrations of α-phellandrene up to 30% v/v in a two-phase system (Heyen 1999). The proteome of *C. defragrans* grown on α-phellandrene revealed the increased expression of the putative RND transporter AmeD, as well as AmeABC, whose genes (*ameABC*) are encoded directly upstream of *ameD* (Petasch et al. 2014). In the same study, a transposon insertion in *ameB* resulted in defective growth on several monoterpenes. The gene cassette *ameABCD* is co-located with several genes associated to the monoterpene metabolism (Fig. 1). In this study, we characterized the AmeABCD system conducting transport and growth studies with *C. defragrans* 65Phen and a deletion mutant lacking the genes *ameABCD*.

## Materials and methods

### Bacterial strains and culture conditions

An in-frame deletion mutant lacking the gene cassette *ameABCD* (*C. defragrans* 65Phen Δ*ameABCD*) was prepared from *C. defragrans* 65Phen Rif^R^ as previously described (Lüddeke et al. 2012), and kindly provided by Jan Petasch (Max Planck Institute for Marine Microbiology, Bremen). *C. defragrans* 65Phen Rif^R^ is referred to in the text as the wild-type strain. The construct for *ameABCD* deletion was prepared using the primer pairs Ameup_*Xba*I_F (ATCGATCTAGATGGCGCGAGGTGGTGTTGTC) and Ameup_*Spe*I_R (CCGACGACTAGTGGCAAGACCCGCAACCTGTG), and Amedown_*Spe*I_F (AAGCTAACTAGTCATGTGTGTCTCCTCTGTGGTT) and Amedown_*Hin*dIII_R (ACTCAAAGCTTCTACTGAAAAACAGGAACGCAG). The deletion mutant and the rifampicin-resistant *C. defragrans* 65Phen (in the text referred to as wild-type) were cultivated in liquid artificial fresh water (AFW) medium under anoxic denitrifying conditions as described elsewhere (Petasch et al. 2014). When indicated 10 or 20 mM of sodium acetate were added as carbon source. Monoterpenes (> 90% purity, Sigma-Aldrich, Germany) were supplied either in the carrier phase 2,2,4,4,6,8,8-heptamethylnonane (HMN) or dissolved in dimethyl sulfoxide (DMSO). Cultures were incubated at 28 °C under constant agitation (60 rpm). Microbial growth was monitored by measuring the optical density at 600 nm.

### Fluorometric assays

As a proof of concept, Nile Red accumulation and extrusion was tested in *C. defragrans* cells by modifying previously described protocols (Bohnert et al. 2010, 2011). Briefly, cells of wild-type *C. defragrans* and Δ*ameABCD* were grown to late exponential phase in AFW medium containing both limonene (3 mM in HMN) and acetate (10 mM) as carbon sources. Cells of both strains grown only on acetate (10 mM) were also tested. The cells were harvested at 5000×*g* for 30 min at 20 °C and washed two times with AFW medium without any organic carbon source. After centrifugation, cells were resuspended in the same carbon-deprived medium to OD_600_ 0.5 and, when indicated, carbonyl cyanide *m*-chlorophenylhydrazone (CCCP) and phe-arg β-naphthylamide (PAβN) were added to final concentrations of 2 µM and 20 µg mL^−1^ (38.5 µM), respectively (from stock solutions of 200 µM CCCP and 2 mM PAβN in DMSO). The handling and preparation of microbial suspensions and chemical solutions was performed in an anaerobic chamber at 4 °C. For influx assays, 198 µL of each cell suspension was transferred to a black 96-well plate (Fluotrac, Greiner Bio-One GmbH, Frickenhausen, Germany). Fifteen minutes after CCCP addition, Nile Red was added to a concentration of 2 µM (from a 200 µM stock solution in DMSO) and homogenized by repeated pipetting. The plate was covered with a sealing-film (thickness 50 µm, Carl Roth GmbH, Karlsruhe, Germany) to minimize exposition to oxygen during fluorescence monitoring. Fluorescence intensity was measured at room temperature with an Infinite M200 PRO (Tecan Austria GmbH, Grödig, Austria) with excitation at 552 nm and emission at 636 nm. Prior to each measurement, the plate was automatically shaken at 691 rpm for 30 s at an amplitude of 1.5 mm. To measure the efflux of Nile Red, cells were incubated anaerobically under constant shaking (60 rpm) with 2 µM of Nile Red and 2 µM of CCCP for 2 h at room temperature. Cells were washed two times by centrifugation at 5000×*g* for 15 min at 20 °C and resuspended in AFW medium deprived of carbon sources. When indicated, PAβN (38.5 µM) was added to cell suspensions. 190 µL of the cell suspension were transferred to a 96-well plate and reenergized with 50 mM of sodium acetate. The plate was covered with sealing film, rapidly taken out of the anaerobic chamber and fluorescence was measured.

### Bioinformatics analysis

NCBI, UniProt and RAST (Overbeek et al. 2014) were used to retrieve protein sequences for AmeABCD and related proteins, and to perform similarity and identity searches (Altschul et al. 1990), and conserved domain architecture analysis (Marchler-Bauer et al. 2017). AmeABCD sequences were analyzed for signal peptides, transmembrane helices and subcellular localization prediction using SignalP v4.1 (Nielsen 2017), TMHHM v2.0 (Krogh et al. 2001) and PSORTb v3.0.2 (Yu et al. 2010), respectively. The results obtained were validated by comparison with the results from InterPro (Finn et al. 2017). Visualization of transmembrane regions was generated with TMRPres2D (Spyropoulos et al. 2004). Three dimensional protein modeling was conducted with Phyre2 (Kelley et al. 2015). For the phylogenetic analysis of AmeD, sequences from the RND families HAE1, HAE2 and HAE3 were extracted from the TCDB database (Saier et al. 2016) and aligned with MAFFT v7.0 (Katoh et al. 2017). A maximum likelihood tree based on the JTT matrix model was calculated using MEGA v7.0 (Kumar et al. 2016) performing 1000 bootstrap replicates. Gene cassettes homologous to *ameABCD* in composition and organization were identified and selected from RAST (Overbeek et al. 2014), UniProt and NCBI databases. Homologous proteins from each cassette were clustered and aligned. The alignments were concatenated into a consensus alignment and used for inferring a maximum likelihood tree. The trees were visualized with Archaeopteryx v0.9921 beta (Han and Zmasek 2009).

## Results and discussion

### Growth on monoterpenes

**Fig. 2:**
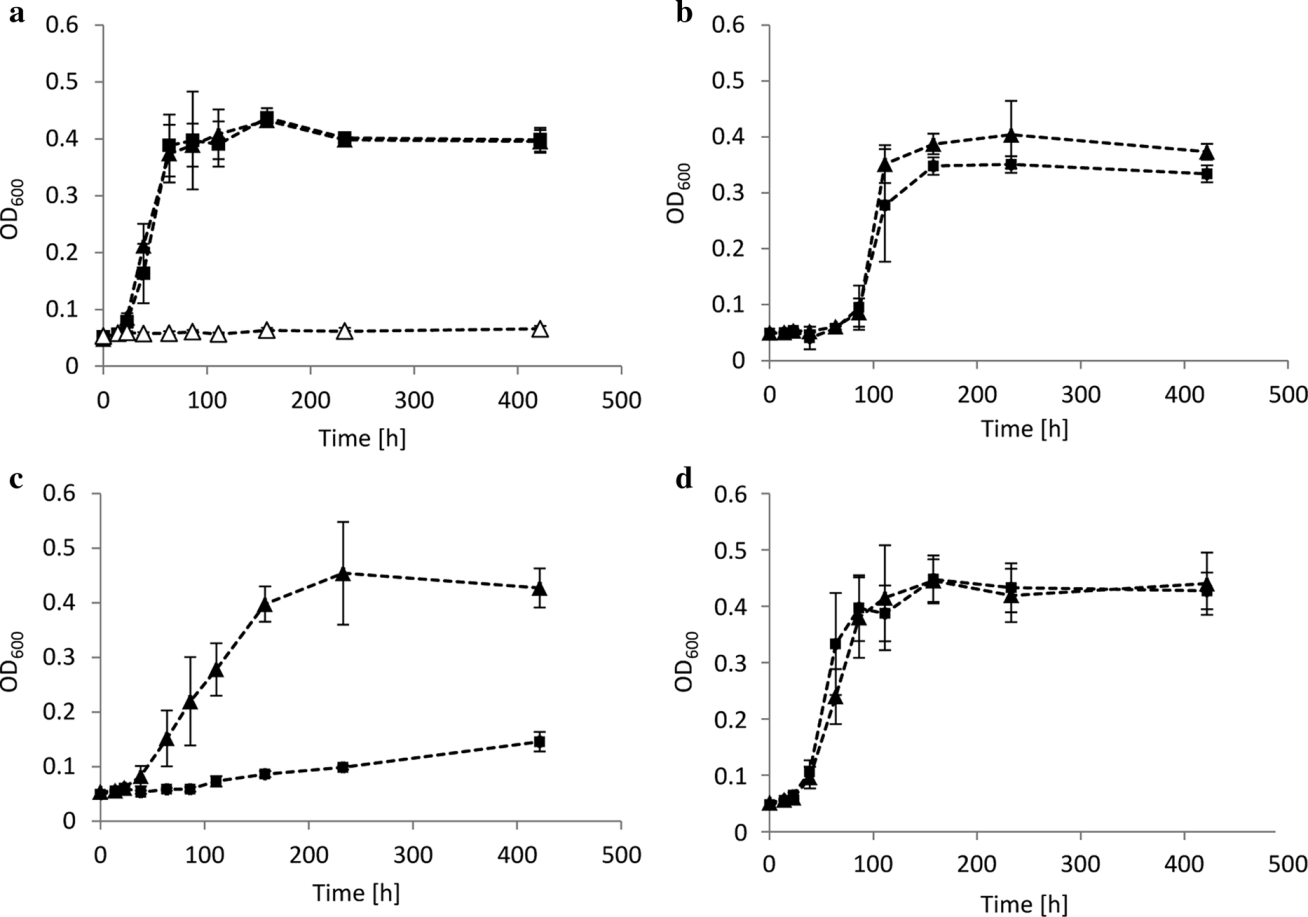
Bacterial growth of wild-type (filled triangles) and Δ*ameABCD* (filled squares) strains of *C. defragrans* 65Phen on acetate (**a**), perillyl alcohol (**b**), limonene (**c**), and acetate and limonene (**d**) monitored at OD_600_. No increase in optical density was observed in cultures deprived of a carbon source (open triangles). Monoterpenes and acetate were added at concentrations of 3 and 10 mM, respectively. The error bars correspond to the standard deviation of at least three independent experiments

The transposon insertion mutant *C. defragrans* 65Phen *ameB*::Tn5 revealed reduced growth on several monoterpenes (Petasch et al. 2014). To assess the role of the putative RND transporter during monoterpene utilization, the wild-type strain and the deletion mutant 65Phen Δ*ameABCD* were compared in growth experiments (Fig. 2). Both strains exhibited similar growth when fed with acetate as sole carbon source (Fig. 2a) as well as with the monoterpenoids perillyl alcohol, perillyl aldehyde and perillic acid (Figs. 2b, S1a, b). The physiological consequences of *ameABCD* deletion were observed during growth on limonene, α-terpinene and other non-functionalized monoterpenes as sole carbon and energy sources (Figs. 2c, S1c–f). In these cultures, the deletion mutant grew only poorly in comparison to wild-type cells, suggesting a reduced tolerance towards non-functionalized monoterpene hydrocarbons. This apparent cytotoxicity is usually the result of monoterpene accumulation in cellular membranes, which affects membrane stability, impairs the maintenance of a proton gradient and hinders energy conservation (Segura et al. 2012; Sikkema et al. 1995). Remarkably, the deleterious effects of limonene on the growth of the deletion mutant were overcome when acetate and limonene were both added to the culture medium (Fig. 2d).

**Fig. 3:**
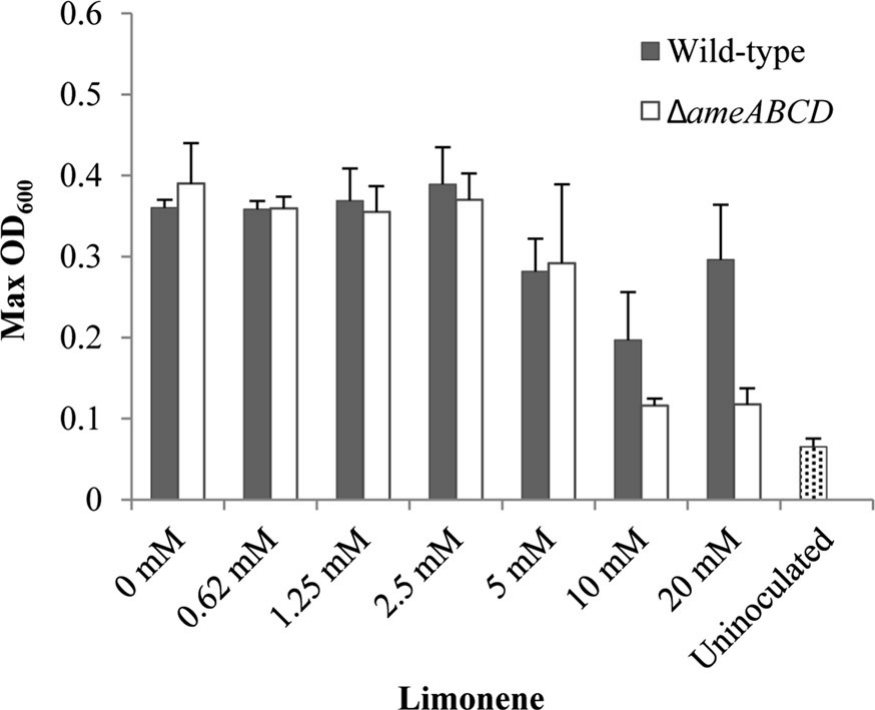
Maximum optical densities of wild-type and Δ*ameABCD C. defragrans* 65Phen in acetate (10 mM) cultures containing limonene at various concentrations. Limonene was dissolved in DMSO prior to addition to the medium to facilitate mass transfer. The error bars indicate the standard deviation of the means for three independent experiments

To further characterize this latter finding, the carrier phase HMN was replaced by DMSO. DMSO facilitated limonene dissolution in the medium and allowed to test higher monoterpene concentrations, while acetate was kept constant (10 mM). Here, the deletion mutant strain showed growth comparable to the wild-type when limonene was added to concentrations up to 5 mM (Figs. 3, S2). The addition of higher limonene concentrations, however, reduced significantly the growth of Δ*ameABCD* cells. These observations suggest that the addition of acetate as cometabolic substrate translates into a significant increase in monoterpene tolerance. It is likely that, as observed in the past, the energy derived from acetate oxidation is used to fuel cellular detoxification mechanisms (Abrahim et al. 2003; Segura et al. 2012; Sikkema et al. 1995; Uribe et al. 1984). Unlike the mutant Δ*ameABCD*, the wild-type strain showed growth in all limonene concentrations tested (Figs. 3, S2), suggesting that proteins AmeABCD are essential for growth under high monoterpene concentrations and may constitute a defense mechanism against monoterpene toxicity in *C. defragrans*. AmeABCD may supplement other monoterpene tolerance mechanisms known in *C. defragrans* such as adaptational changes in membrane composition and biotransformation of monoterpene substrates (Foss and Harder 1998; Harder and Marmulla 2017).

### Influx and efflux of Nile Red

**Fig. 4:**
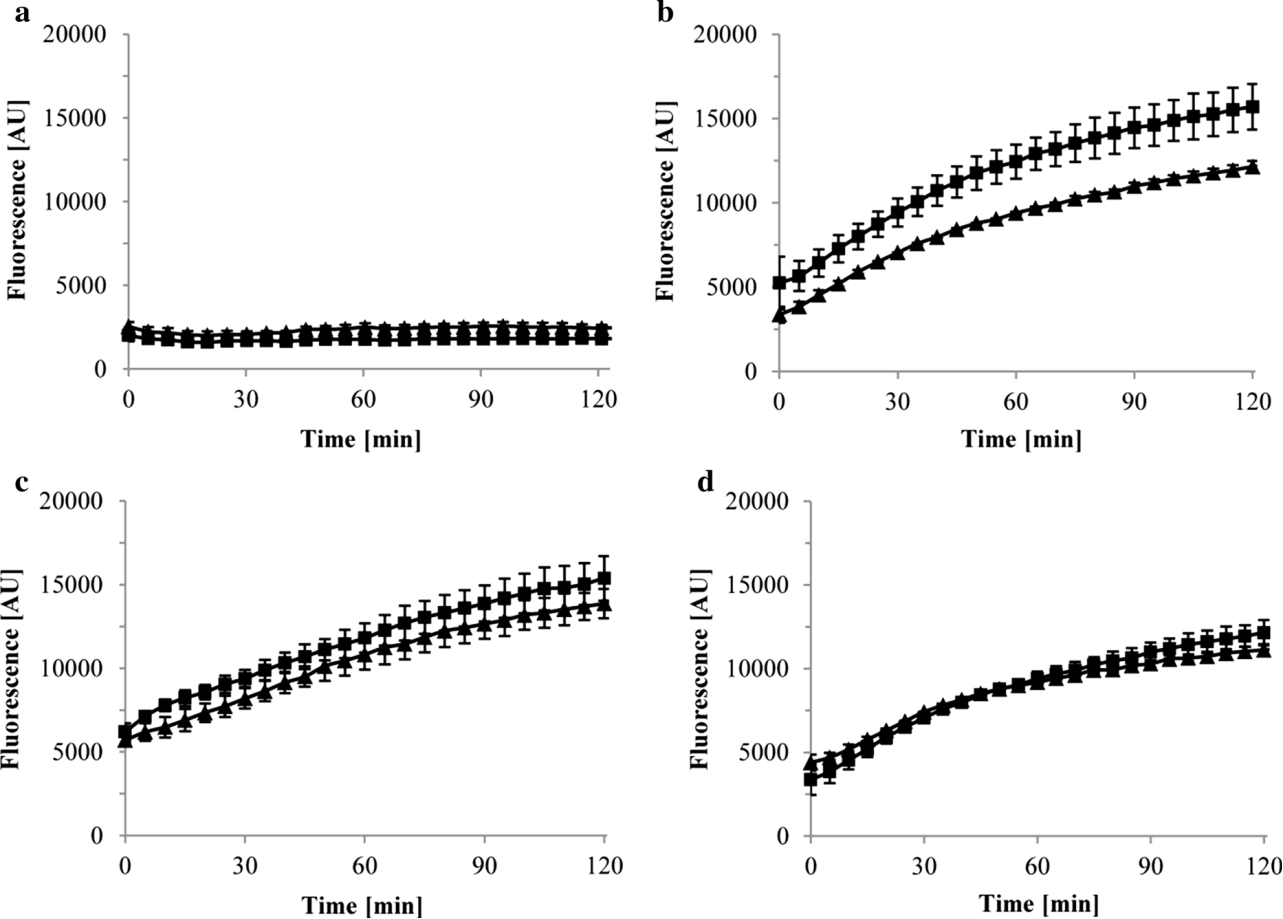
Influx of Nile Red in cells of *C. defragrans* 65Phen grown on acetate (10 mM) in cometabolism with limonene (3 mM) (wild-type: filled triangles, Δ*ameABCD*: filled squares). Nile Red (2 µM) was added directly to the cells (**a**) or together with 2 µM of the proton-gradient uncoupling agent carbonyl-cyanide *m*-chlorophenylhydrazone (CCCP) (**b**). The combined effect of CCCP and the RND pump inhibitor phe-arg β-naphthylamide (PAβN, 38.5 µM) is also shown (**c**). Cells grown on acetate as only carbon and energy source were also treated with CCCP and tested for Nile Red accumulation (**d**). The influx of Nile Red was followed by measuring fluorescence intensity (excitation 552 nm, emission 636 nm). The error bars indicate the standard deviation of the means for at least three independent experiments

A set of fluorometric assays were conducted to monitor the in vivo accumulation and export of Nile Red in *C. defragrans* wild-type and Δ*ameABCD*. The cells were grown in AFW medium with 10 mM acetate as only carbon source and in cometabolism with 3 mM limonene dissolved in HMN. Given its lipophilic nature, Nile Red accumulates in cell membranes which results in a significant increase in its fluorescent quantum yield (Blair and Piddock 2016). Assays with Nile Red and other environment-sensitive dyes are routinely used as proxy to show the contribution of efflux pumps in bacterial resistance to xenobiotics (Blair and Piddock 2016; Paulsen et al. 1996; Soto 2013). In our experiments Nile Red accumulated in *C. defragrans* cells treated with the protonophore carbonyl cyanide *m*-chlorophenyl hydrazine (CCCP), but not in untreated cells (Fig. 4). The lack of fluorescence signal in the untreated controls (Fig. 4a) indicates that Nile Red is a suitable substrate for proton-driven efflux pumps present in physiologically active cells of both the wild-type and the Δ*ameABCD* strains. When de-energized with CCCP, the mutant Δ*ameABCD* exhibited a slightly higher Nile Red accumulation than the wild-type (Fig. 4b), suggesting a contribution of AmeABCD to a residual export of Nile Red in the wild-type strain even in the presence of the protonophore. The addition of the RND transporter inhibitor phenylalanine-arginine β-naphthylamide (PAβN) increased the accumulation of Nile Red in the wild-type strain and thus confirmed that the residual export activity was caused by still active RND efflux pumps (Fig. 4c). Interestingly, when both strains were grown on acetate as only carbon and energy source, they once more accumulated similar amounts of Nile Red (Fig. 4d). The latter coincides with previous observations which showed that the expression of genes *ameABCD* in *C. defragrans* is triggered by exposure to monoterpenes, but not detectable when cells are grown on acetate only (Petasch et al. 2014).

**Fig. 5:**
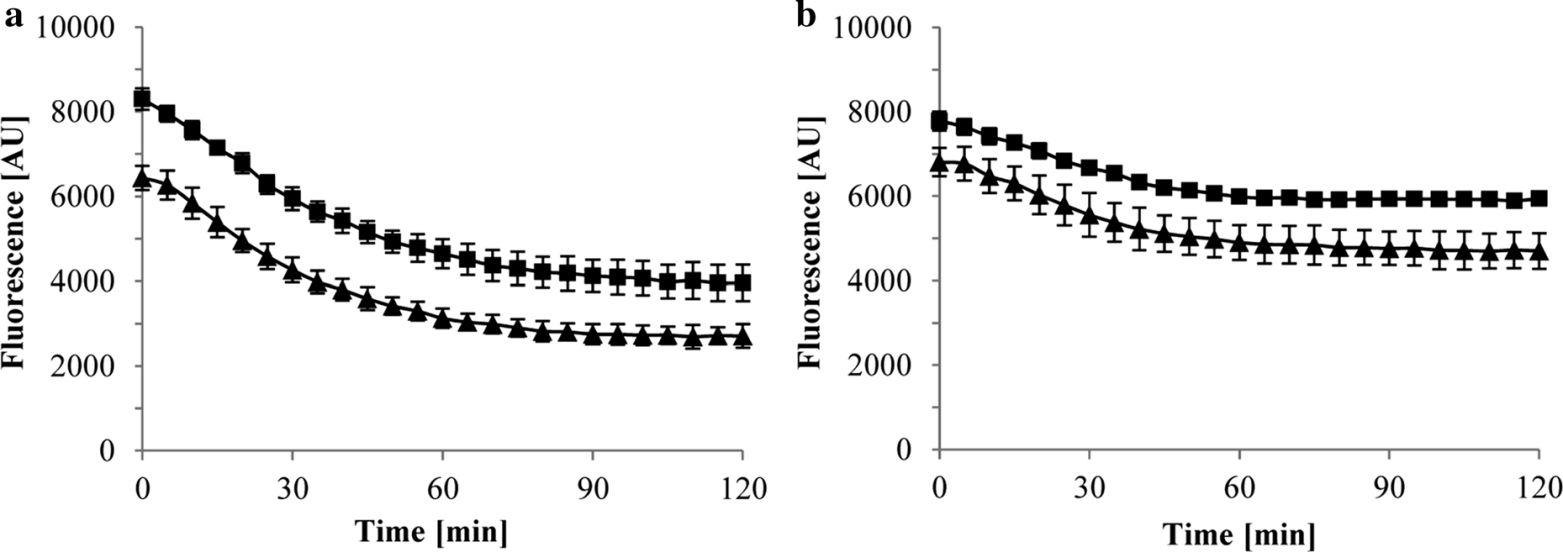
Nile Red efflux by wild-type (filled triangles) and Δ*ameABCD* (filled squares) strains of *C. defragrans* 65Phen. Bacterial cells were preloaded with 2 µM Nile Red in the presence of CCCP (2 µM) at 28 °C under constant shaking (120 rpm) for 2 h. After two washing steps, the cells were reenergized with 50 mM acetate and fluorescence was recorded (**a**). The effect of 38.5 µM of PAβN on reenergized cells was tested (**b**). The error bars indicate the standard deviation of the means for at least three independent experiments

Real-time efflux experiments typically require the re-energization of CCCP-treated cells loaded with Nile Red using a readily fermentable substrate such as glucose (Bohnert et al. 2010; Paixao et al. 2009). In non-fermenting bacteria such as *C. defragrans* dye efflux is generally inferred from measuring intracellular dye accumulation rather than from online efflux measurements (Morita et al. 2001; Richmond et al. 2013). Nevertheless, as a proof of concept a real-time Nile Red efflux assay was conducted with Nile Red-preloaded *C. defragrans* wild-type and Δ*ameABCD*. After Nile Red loading, acetate (50 mM) was added to re-energize the cells and fluorescence was recorded. The efflux of Nile Red showed almost identical apparent kinetics in both strains (Fig. 5a). In experiments where no acetate was added similar efflux curves were observed (data not shown), indicating that acetate addition has no effect on Nile Red efflux in *C. defragrans*. The observed decrease in fluorescence may be caused by passive diffusion of Nile Red to the outside of the cells or by reactivation of transporters after the removal of CCCP during the intermediate washing steps. The obligate-respiring *C. defragrans* reenergizes slowly with acetate in comparison to fermenting bacteria. The latter restore active dye efflux within seconds after glucose addition (Blair and Piddock 2016; Bohnert et al. 2011; Iyer et al. 2015). We used the addition of PAβN to wild-type and Δ*ameABCD* cells to assess the involvement of active exporters on the efflux process. Here, the extrusion of Nile Red was reduced in both strains (Fig. 5b), suggesting at least a partial contribution of RND pumps in this process.

### In silico analysis of RND efflux transporters in *C. defragrans*

The genome of *C. defragrans* contains nine putative RND pumps. Seven of them (NCBI Acc. Numbers CDM22941, CDM23198, CDM23199, CDM24125, CDM24282, CDM24412 and CDM25333) affiliate to the HAE1 family, the most studied group of RND transporters. HAE1 transporters associate with periplasmic and outer membrane proteins and mostly confer tolerance to xenobiotics in clinical and environmental isolates (Blanco et al. 2016; Tseng et al. 1999).

Another transporter (CDM25549) belongs to the SecDF family, a group of chaperon transporters involved in the export of proteins across the inner membrane (Tsukazaki and Nureki 2011). The ninth protein, AmeD (CDM25262), affiliates with the HAE3 family, a family of mainly uncharacterized transporters.

AmeD along with AmeABC were up-regulated in the proteome of α-phellandrene-grown *C. defragrans* (Petasch et al. 2014). The closest homologs for the cluster AmeABCD were the hypothetical proteins EPZ15054, EPZ15055, EPZ15056 and EPZ15057 from *Thauera terpenica* 58Eu^T^ with sequence identities ranging between 61 and 80%. Similar to *C. defragrans*, *T. terpenica* 58Eu^T^ is able to anaerobically mineralize a wide range of monocyclic and bicyclic monoterpenes (Foss and Harder 1998).

**Fig. 6:**
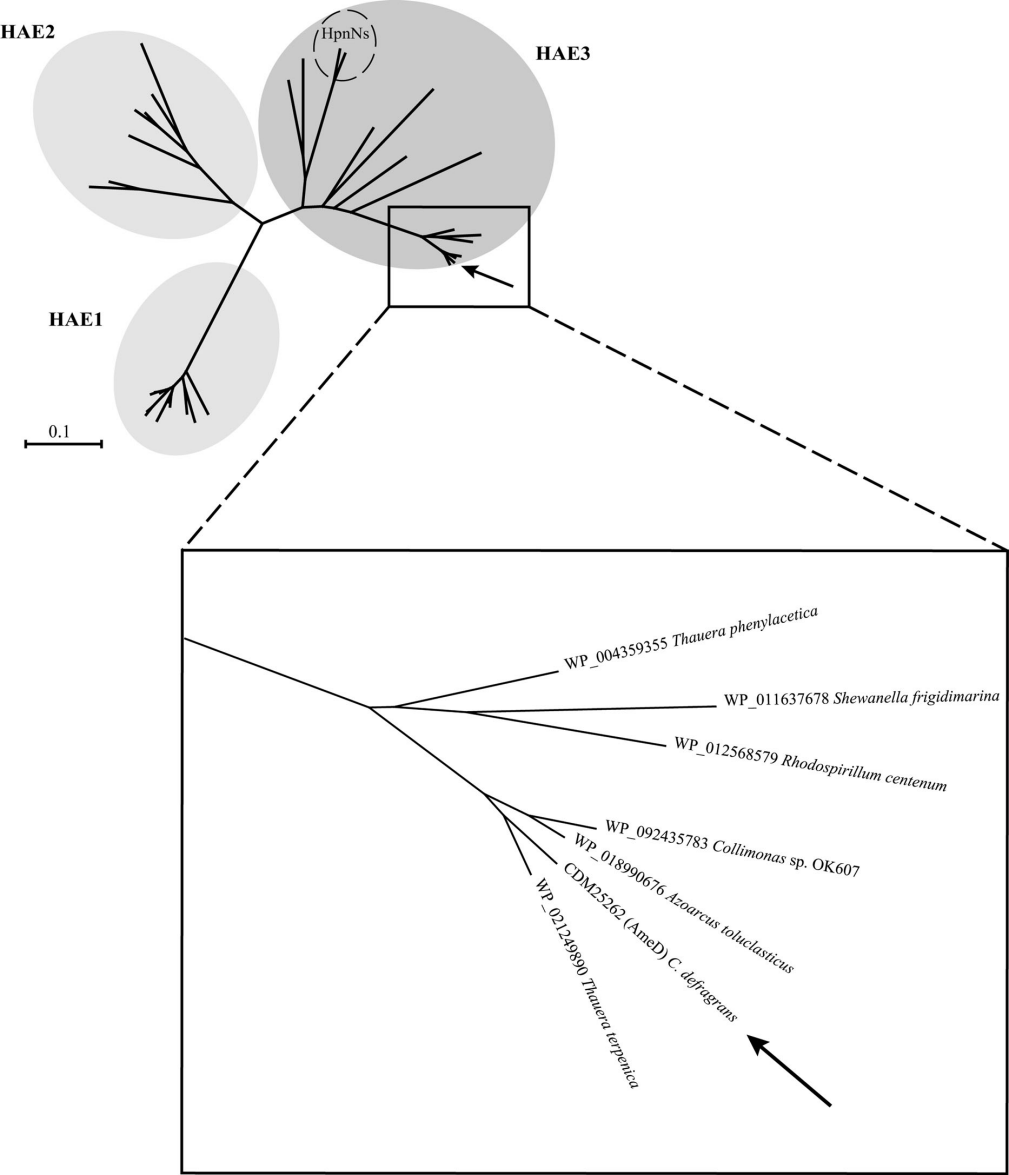
Unrooted phylogenetic tree of representatives from the RND families HAE1, HAE2 and HAE3. AmeD (arrow) and its closest homologs clustered in a distinct branch within HAE3 (square). RND hopanoid transporters (HpnN) from *Rhodopseudomonas palustris* TIE-1 and *Burkholderia multivorans* are indicated within the dashed circle. Enlarged area displays the sequences most closely related to AmeD within the HAE3 family. Proteins sequences of each HAE family were aligned using MAFFT (Katoh et al. 2017). A maximum likelihood tree was calculated with MEGA 7 (Kumar et al. 2016) and visualized with Archaeopteryx 0.9921 beta (Han and Zmasek 2009)

**Fig. 7:**
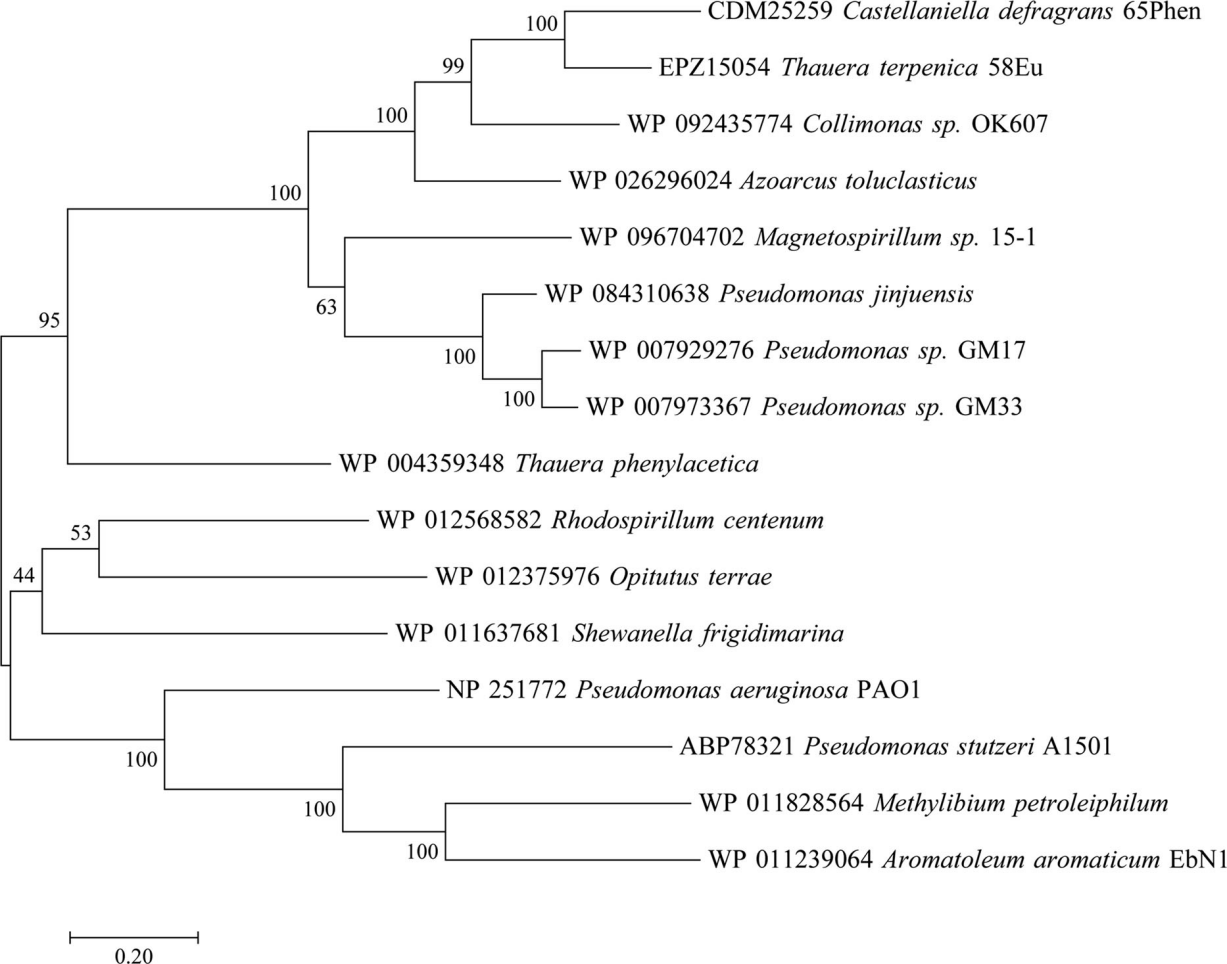
Unrooted maximum likelihood tree based on JTT matrix-based model inferred from concatenated protein sequence alignments with homology to *C. defragrans* AmeABCD. Gene cassettes were identified and downloaded from RAST (Overbeek et al. 2014), NCBI and UniProt. Homologous proteins from each cassette were clustered and aligned using MAFFT (Katoh et al. 2017). The alignments were concatenated and used for calculating a maximum likelihood tree with MEGA 7 (Kumar et al. 2016). The tree was visualized with Archaeopteryx 0.9921 beta (Han and Zmasek 2009)

AmeA (CDM25259) is a protein of 605 amino acid residues. It consists of an N-terminal signal peptide and a large conserved domain of unknown function (DUF1302). Its predicted localization as an outer membrane protein concurs with its inferred structural homology with the outer membrane protein OpcA (PDB: 2VDF) from *Neisseria meningitidis* (Cherezov et al. 2008; Moore et al. 2005). The second up-regulated protein—AmeB (CDM25260, 451 aa)—was predicted as periplasmic and affiliated to the LolA superfamily (DUF1329). Although the crystal structure of several proteins within this superfamily have been resolved (e.g. PDB: 4Z48, 3BK5 and 3BUU), for most their function in the periplasm remains unknown. Protein AmeB has been shown to be essential for growth on monoterpenes since a transposon insertion within *ameB* resulted in reduced growth on limonene, myrcene and perillic acid (Petasch et al. 2014). Protein AmeC (CDM25261, 316 aa) affiliates to the COG4447, a group of proteins related to stability and assembly factors of the photosystem II in plants. AmeC is also predicted as a periplasmic protein and hence is likely involved in the assembly of the RND transporter complex. Lastly, protein AmeD (CDM25262, 787 aa) belongs to the RND efflux transporter superfamily and affiliates with the hydrophobe/amphiphile efflux-3 family (HAE3) (COG1033). The 12 transmembrane-spanning (TMS) regions and two periplasmic loops (located between TMS 1 and 2 and between TMS 7 and 8) conserved among all RND efflux pumps were predicted from the amino acid sequence (Fig. S3) (Paulsen et al. 1996; Tseng et al. 1999). In a phylogenetic reconstruction with sequences from the RND transporter families HAE1, HAE2 and HAE3, AmeD and its closest homologs clustered in a distinct lineage within the HAE3 branch (Fig. 6). To date, the only characterized representatives from HAE3 are two closely related hopanoid transporters (HpnN) from *Rhodopseudomonas palustris* TIE-1 and *Burkholderia multivorans* (Doughty et al. 2011; Kumar et al. 2017). In these two oth organisms, HpnN catalyzes the translocation of hopanoids from the inner membrane to the periplasm without the need for association or co-transcription with periplasmic proteins or outer membrane channels. In contrast, all members in the AmeD lineage are encoded within gene cassettes composed of outer- and inner-membrane proteins homologous to AmeA and AmeD, respectively, and two periplasmic proteins homologous to AmeB and AmeC. Both the composition and the organization of such gene cassettes are conserved among the members of this lineage, sharing a close overall phylogenetic history (Fig. 7), and likely representing a new subfamily within HAE3. Intriguingly, this subunit composition and architecture resembles that of RND transporters of the HAE1 family described in numerous Gram-negative bacteria (Daury et al. 2016). Although most HAE1 transporter complexes are composed of three proteins, a few examples for the requirement of a fourth protein are known. The transporter systems CusCFBA and TriABC-OpmH both require two periplasmic proteins to catalyze the efflux of heavy metals (i.e. Cu^+^ and Ag^+^) and the antimicrobial triclosan, respectively (Delmar et al. 2014; Mima et al. 2007). Additional efflux systems such as the MuxABC-OpmB and AcrABZ-TolC also require a forth protein which locates in the inner membrane and in the cytoplasm, respectively (Mima et al. 2009; Wang et al. 2017). In our case, the roles of AmeB and AmeC cannot be predicted unequivocally and their definition await for the construction and experimentation with corresponding in-frame, non-polar deletion mutants. Nonetheless, the affiliation of both proteins with periplasmic chaperons suggests their participation in the assembly and stability of the RND complex and hence both may act as MFPs.

## Conclusion

In this study, the role of the putative RND transporter complex AmeABCD in monoterpene growth of *C. defragrans* was investigated. The results showed reduced biomass yield in an *ameABCD* deletion mutant growing on non-functionalized monoterpenes as sole carbon sources. The addition of acetate as cometabolic substrate increased significantly the tolerance of the deletion mutant towards limonene. Deletion of *ameABCD* resulted also in higher net influx of Nile Red into CCCP-treated *C. defragrans*, suggesting the participation of the RND transporter in dye efflux in the wild-type cells. AmeD is affiliated to a lineage of RND transporters within the HAE3 family that unlike other HAE3 members associate with two periplasmic proteins and one outer membrane channel analogous to some RND transporter systems of the HAE1 family. It is suggested that AmeB and AmeC act both as periplasmic MFPs. Their homology to chaperones and assembly factors grant both proteins with potential for facilitating protein–protein interactions and hence the assembly of the RND complex. The inducible proteins AmeABCD provide *C. defragrans* with tolerance against the toxic monoterpenes that it naturally uses as carbon and energy sources.

## Electronic supplementary material

Below is the link to the electronic supplementary material.

Supplementary material 1 (PDF 729 kb)
