# Supplementary material for: An RND transporter in the monoterpene metabolism of Castellaniella defragrans
Source: Biodegradation. 2018 Oct 17;30(1):1–12. doi: 10.1007/s10532-018-9857-6 (PMC6394551; doi:10.1007/s10532-018-9857-6)
Supplement: Supplementary file 1 — Supplementary material 1 (PDF 729 kb) [file 10532_2018_9857_MOESM1_ESM.pdf]

Supplementary material

An RND transporter in the monoterpene metabolism of *Castellaniella defragrans*

Edinson Puentes-Cala*, Jens Harder

Dept. of Microbiology, Max Planck Institute for Marine Microbiology, Bremen

*To whom correspondence should be addressed: Edinson Puentes-Cala, Dept. of Microbiology, Max Planck-Institute for Marine Microbiology, Celsiusstr. 1, D-28359 Bremen, Germany, Tel.: +49 421 2028-748; Fax: +49 421 2028-790; E-mail: epuentes@mpi-bremen.de; epuentes@corrosion.uis.edu.co. ORCID ID: 0000-0003-0587-2177.

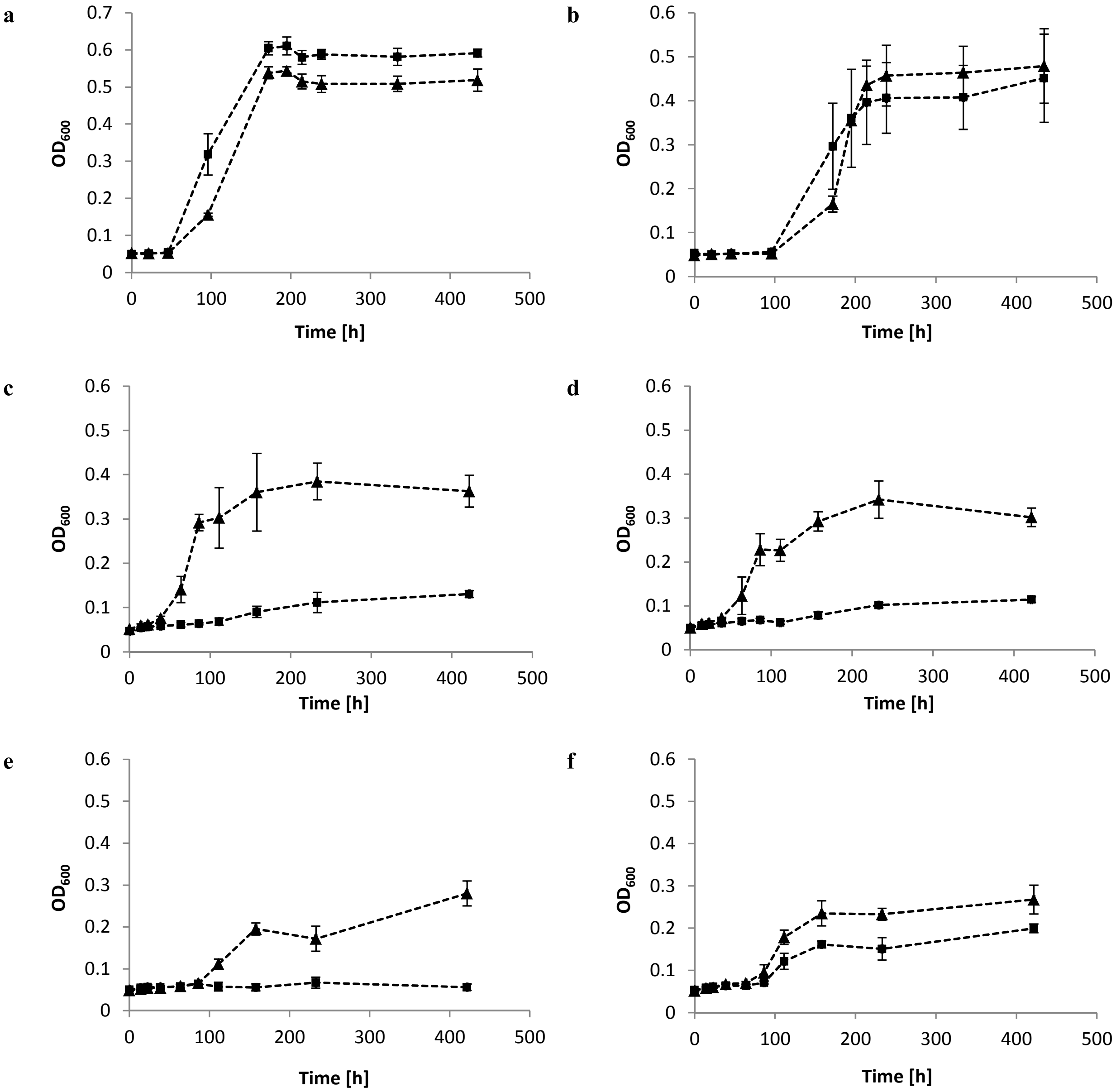

Fig. S1 Bacterial growth of wild-type (▲) and Δ*ameABCD* (■) strains of *C. defragrans* 65Phen on perillyl aldehyde (a), perillic acid (b), α-terpinene (c), terpinolene (d), sabinene (e), and myrcene (f). Monoterpenes were added in a carrier phase (HMN) to a final concentration of 3 mM. The error bars indicate the standard deviation of the means for three independent experiments

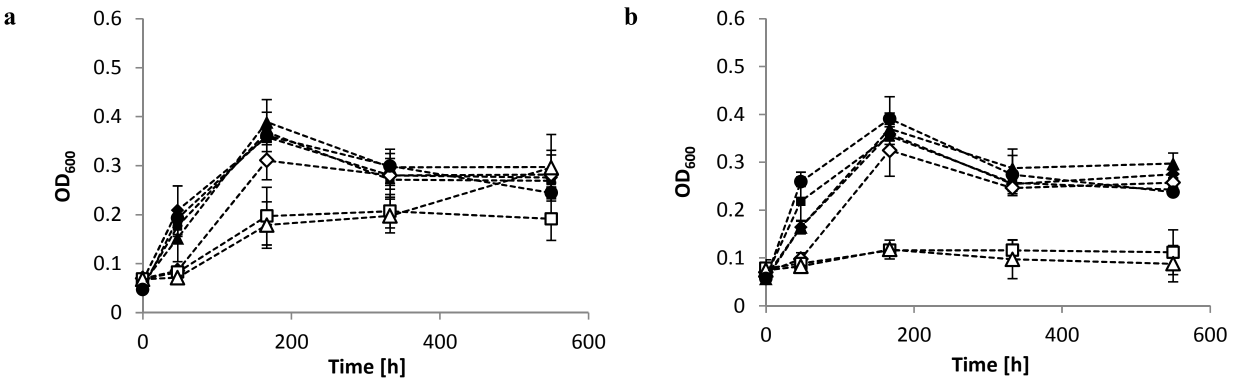

Fig. S2 Growth of wild-type (a) and Δ*ameABCD* (b) *C. defragrans* 65Phen in acetate (10 mM) in cometabolism with various concentrations of limonene: 0 mM [●], 0.62 mM [♦], 1.25 mM [■], 2.5 mM [▲], 5 mM [◊], 10 mM [□], 20 mM [Δ]. To enhance mass transfer limonene was predissolved in DMSO. The error bars indicate the standard deviation of the means for three independent experiments

a

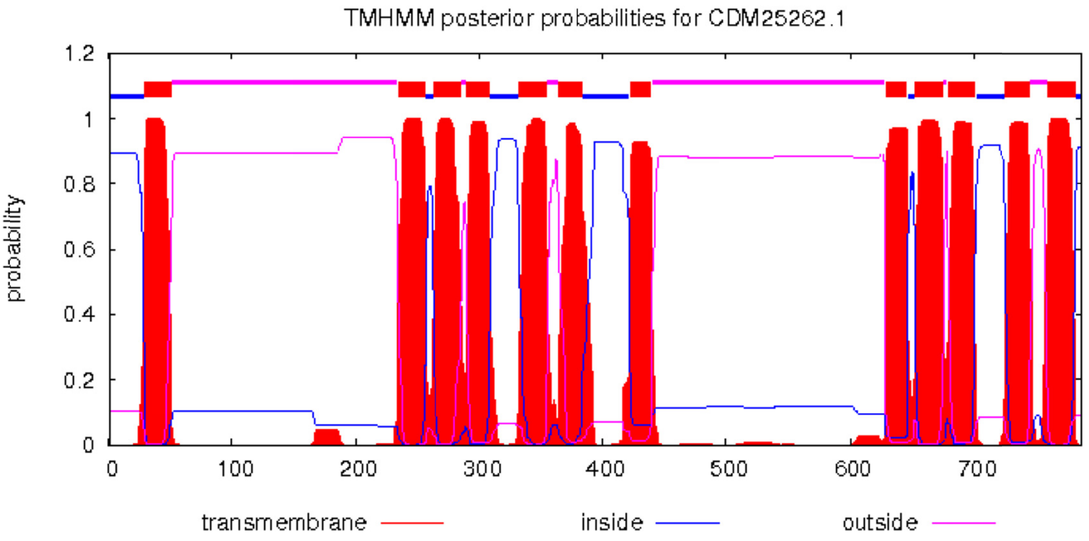

b

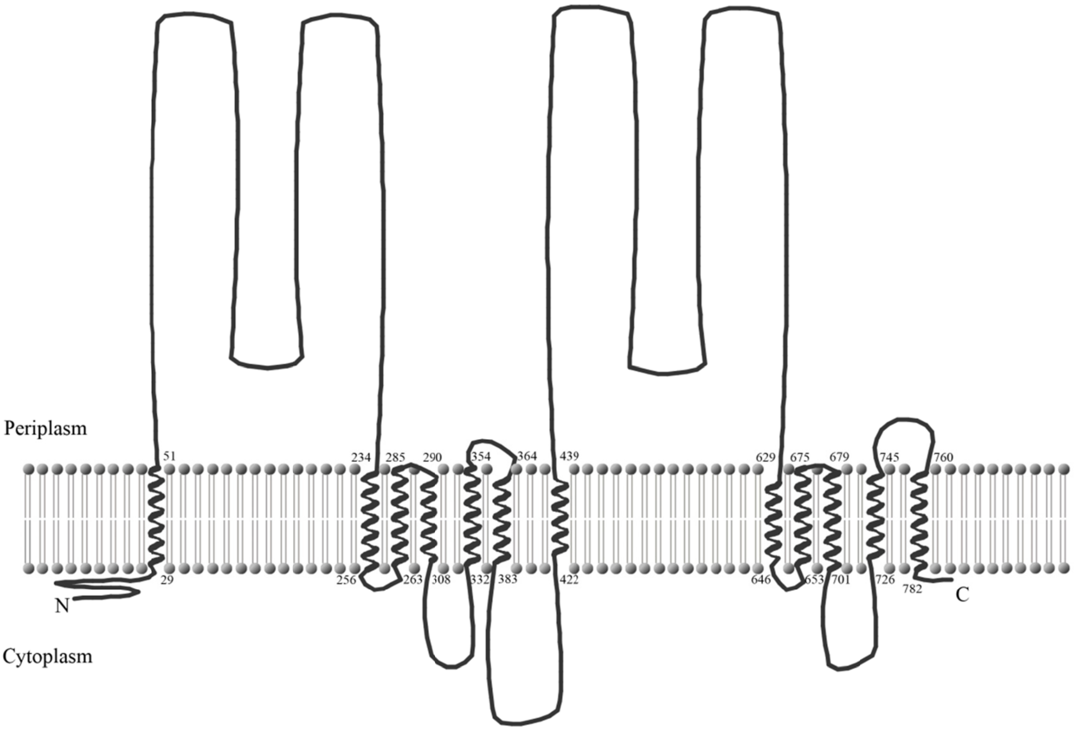

Fig. S3 Transmembrane-spanning regions predicted in AmeD using TMHHM ver. 2.0 (Sonnhammer *et al*. 1998) (a) and visualized by TMRPres2D ver 0.93 (Spyropoulos *et al*. 2004) (b)
